# A community-based intervention to prevent serious complications and death 2 years after discharge in people with spinal cord injury in Bangladesh (CIVIC): a randomised trial

**DOI:** 10.1038/s41393-020-00546-9

**Published:** 2020-09-11

**Authors:** Mohammad Sohrab Hossain, Lisa A. Harvey, Md. Shofiqul Islam, Md. Akhlasur Rahman, Stephen Muldoon, Fin Biering-Sorensen, Stephen Jan, Hueiming Liu, Qiang Li, Ian D. Cameron, Valerie Taylor, Richard I. Lindley, Laurent Billot, Robert D. Herbert

**Affiliations:** 1 Centre for the Rehabilitation of the Paralysed, Chapain, Savar, Dhaka, 1343 Bangladesh; 2 John Walsh Centre for Rehabilitation Research, University of Sydney, Kolling Institute, Royal North Shore Hospital, St Leonards, 2065 NSW Australia; 3 Muldoon Rehabilitation, 72 Liscreevin Road, Lisnarick, Co Fermanagh, BT94 1PZ Northern Ireland; 4 Department for Spinal Cord Injuries, University of Copenhagen, Havnevej 25, DK-3100 Hornbæk, Denmark; 5 The George Institute for Global Health, Faculty of Medicine, University of New South Wales, PO Box M201, Missenden Road, Camperdown, 2050 NSW Australia; 6 Westmead Applied Research Centre, University of Sydney, Sydney, NSW Australia; 7 Neuroscience Research Australia (NeuRA), Barker Street, Randwick, 2031 NSW Australia

**Keywords:** Public health, Neurology

## Abstract

**Study design:**

Randomised controlled trial.

**Objectives:**

To determine the effectiveness of a sustainable community-based intervention designed to prevent serious complications and death 2 years after discharge in people with spinal cord injury in Bangladesh.

**Setting:**

Bangladesh.

**Methods:**

A pragmatic randomised controlled trial was undertaken. People who had sustained a spinal cord injury in the preceding 2 years, were wheelchair-dependent, and were about to be discharged from hospital in Bangladesh were recruited and randomised to an Intervention or Control group using a concealed allocation procedure stratified by level of lesion (tetraplegia/paraplegia). Participants in the Intervention group received 36 phone calls and three home visits over the first 2 years following discharge. All participants received usual post-discharge care. Survival status and date of death were determined by blinded assessors 2 years after randomisation.

**Results:**

Between July 2015 and March 2018, 410 participants were randomised (204 to Intervention, 206 to Control). There was no loss to follow up. At 2 years, 15 (7.4%) participants in the Intervention group and 16 (7.8%) participants in the Control group had died (hazard ratio from unadjusted Cox model = 0.93 [95% CI, 0.46 to 1.89]; *p* from log rank test 0.85). There were no clinically important or statistically significant average causal effects of intervention on the incidence or severity of complications.

**Conclusion:**

A program of community-based care for people with recent spinal cord injury in Bangladesh involving frequent phone contact and occasional in-person contact with a health professional after discharge from hospital is no better at preventing death at 2 years than usual care.

## Introduction

In low- and middle-income countries, people who sustain spinal cord injuries are likely to experience serious complications after discharge from hospital. Common complications include pressure ulcers, respiratory and urinary tract infections, depression, faecal and urinary incontinence, and autonomic dysreflexia [1, 2]. These complications can be life-threatening [2, 3]. We found that 19% of wheelchair-dependent people with spinal cord injury discharged from a large hospital in Bangladesh were dead within 2 years of discharge [4] and 31% were dead within 5 years [5].

Mortality rates after discharge from hospital in low- and middle-income countries are much higher than in high-income countries [2, 3]. That may be because in most high-income countries structured follow-up programs are used to prevent and manage secondary complications [2, 6]. These programs typically involve regular face-to-face follow-up with clinicians who screen for complications and provide advice and support. In high-income countries, most people with spinal cord injury have ongoing access to medical care and can be hospitalised if required [2, 7, 8]. In contrast, follow-up services are not routinely available in many low- and middle-income countries because the cost of providing such services is prohibitive and because travel to clinics and hospitals can be difficult, particularly for people who live in rural areas.

The first-line strategies for prevention and management of common complications after spinal cord injury are neither expensive nor technically difficult to implement. For example, pressure ulcers can be prevented and managed by providing suitable cushions and mattresses and regular repositioning [9, 10]. Bladder infections can be prevented and managed with clean, regular self-catheterisation and adequate fluid intake [11]. Whilst most of these strategies are not based on the results of high quality trials, they are sensible and recommended in all major guidelines [2, 9, 11, 12]. Presumably the strategies are applicable and implementable in both high- and low-income countries.

In an attempt to reduce the rates of secondary complications and death soon after discharge from hospital, we designed an affordable and potentially sustainable community-based program of care for people who had been discharged from hospital with spinal cord injury. A key feature of the intervention is frequent phone contact between health professionals and people with spinal cord injury in the 2 years after discharge from hospital. The health professionals help people with spinal cord injury identify complications and intervene early, before the complications become severe, and provide advice on simple strategies that people with spinal cord injury can implement themselves to prevent and manage complications.

There is widespread acceptance of the need to provide programs of care for people living with spinal cord injury in low- and middle-income countries [2], but there have been few randomised trials evaluating the effectiveness of those programs. We refined and updated a search conducted as part of a Campbell Systematic Review [13] to identify trials of any type of community-based program for people with spinal cord injury from low- or middle-income countries. The search identified only two trials, both of which were conducted by members of our research team. One trial of 120 participants, conducted in India and Bangladesh, evaluated the effectiveness of a 12-week program of phone-based support for people with spinal cord injury who had developed pressure ulcers [14]. The evidence was suggestive but not confirmatory of a beneficial effect: the intervention reduced pressure ulcer size by, on average, 2.3 cm^2^ (95% CI -0.3 to 4.9). The second trial was a pilot trial of 30 people with spinal cord injury followed for 2 years [15]. It confirmed the feasibility of conducting a large trial of our community-based program of care. The intervention was further refined prior to undertaking the definitive trial. That trial—the CIVIC trial—is reported here.

The purpose of the CIVIC trial was to determine the effectiveness of a community-based program of care involving frequent phone and occasional in-person contact with a health professional after discharge from hospital with spinal cord injury in Bangladesh. We hypothesised that the intervention would prevent serious complications and death in the first 2 years after discharge in this population.

## Methods

### Study design

The CIVIC trial was a pragmatic, assessor-blinded, two-arm, parallel, randomised, superiority trial. The trial protocol and statistical analysis plan have been published [16, 17]. The trial was prospectively registered (ACTRN12615000630516; U1111-1171-1876).

### Participants

Patients admitted to the Centre for the Rehabilitation of the Paralysed (CRP) with a recent spinal cord injury were eligible to participate in the trial if they were at least 15 years of age, were wheelchair-dependent on discharge, had sustained a traumatic or non-traumatic spinal cord injury in the preceding 2 years and provided written consent. The CRP provides specialised inpatient rehabilitation for over 400 people with recent spinal cord injury each year. It accepts patients with recent traumatic and non-traumatic injuries from across Bangladesh irrespective of income. It is the only specialised spinal cord injury centre in Bangladesh and one of the largest rehabilitation centres of its kind. From 12th July 2015, trial staff screened all people with spinal cord injury prior to discharge from hospital.

### Randomisation

Participants were randomised in permuted blocks stratified by level of lesion (tetraplegia or paraplegia). The randomisation schedule was concealed from potential participants, trial staff and all investigators, except an Australia-based investigator (RDH) who generated the allocation schedule and two India-based trial staff who dispensed allocations by email. Participants were approached and enrolled by trial staff but allocation was requested by the site coordinator (MSI). Neither the investigator nor the two trial staff had any involvement in recruitment of trial participants. Each eligible participant was randomised to either an Intervention group or a Control group.

### Blinding

The nature of the intervention precluded blinding of trial participants and the healthcare professionals who administered the intervention. However, the assessors were blinded. To reduce potential for unblinding, assessors were not permitted to share office space or correspond with other trial staff. Assessors were naïve to the nature of the trial intervention and were trained separately to other trial staff. Trial staff did not share information about the trial with CRP staff or patients.

### Procedures

The Intervention group received community-based care in addition to usual care. The Control group received only usual care.

To deliver community-based care, healthcare professionals provided phone-based support to participants fortnightly in the first year and monthly in the second year following discharge from hospital. In addition, a healthcare professional visited each participant and the participant’s family in the home on three occasions: twice in the first year and once in the second year. The health professionals had backgrounds in nursing and physiotherapy.

At each contact (i.e., during each phone call or home visit), participants were screened for pressure ulcers, urinary tract infection, faecal or urinary incontinence, depression, autonomic dysreflexia, and respiratory complications. Where available and appropriate, the camera and video facilities of smartphones were used to help monitor complications. If there was any evidence of a complication, the healthcare professional provided advice to the participant and the participant’s family about management of the complication and then more closely monitored the participant until the complication had resolved. Where necessary and possible, the healthcare professionals referred participants to local service providers (although our process evaluation indicated that these services were either not available or difficult to access [18]). The advice provided to participants followed international clinical practice guidelines [19–21] modified for the Bangladesh context. In addition, healthcare professionals provided education and emotional support. They encouraged the routine implementation of self-help strategies designed to prevent complications, attempted to reduce psychological distress, and encouraged social engagement. They also sought solutions for mobility and self-care limitations. Participants were encouraged to set goals that were regularly reviewed. The healthcare professionals also interacted with and supported participants’ families. At each home visit, the healthcare professionals assessed the participant’s home situation, encouraged the use of cushions and mattresses appropriate for preventing pressure ulcers, and reviewed bladder and bowel care protocols. The home visits were also important for establishing rapport between the health professional and participant and for increasing the health professionals’ understandings of participants’ home environments. On the first home visit, participants in the Intervention group were provided with a pictorial educational booklet specifically designed for the trial. Participants were also provided with health care products such as wound dressings and urinary catheters to a total of AUD80 (~USD51) if they could not otherwise afford these items (see ref. [18] for more details).

Participants in the Control group received only usual care (see ref. [18] for details). In brief, usual care did not include routine post-discharge follow-up. However, CRP staff members sometimes phoned patients to provide advice and support, and occasionally CRP staff visited nearby patients in their homes. On completion of the trial, participants in the Control group reported receiving a median (interquartile range) of 3 (1 to 5) phone calls, 1 (0 to 2) home visit from CRP staff, and 1 (0 to 4) contact with other healthcare professionals over the 2-year study period.

### Outcomes

Data used to characterise the sample were collected at baseline. These included data on age, time since injury, gender, neurological level, type of SCI (traumatic or non-traumatic), American Spinal Injury Association Impairment Scale (AIS), total motor score, marital status, employment status prior to injury, monthly and family income prior to injury, and anticipated primary care giver post discharge.

All outcomes were measured by blinded assessors 2 years after randomisation (there was a ±1-month window for these to be conducted). Initially, the blinded assessor phoned the participant and then travelled to the participant’s home to conduct the assessment. If, however, the assessor was informed at the initial phone contact that the participant had died, the assessor interviewed family members over phone. Some of the secondary outcomes were also assessed at baseline (ie., prior to randomisation, while participants were still in hospital).

The primary outcome was time to death from any cause. The date of death was obtained by asking family members.

Secondary outcomes were burden of complications, prevalence of pressure ulcers, severity of pressure ulcers, depression, participation, quality of life, and independence. The secondary outcomes reflected the prevalence rather than incidence of complications. By measuring prevalence of secondary outcomes at baseline and 2 years rather than monitoring incidence of secondary outcomes over the 2-year period we avoided the need to contact participants in the Control group during the 2-year period. That was desirable because any contact between trial staff and Control group participants during the 2-year period could have caused contamination of the intervention. All questionnaires used to obtain self-reported outcomes were administered in Bangla under the guidance of the assessor.

The burden of complications was measured using the Spinal Cord Injury Secondary Conditions Scale (SCI-SCS) [22]. This is a 16-item scale. Each item is scored from 0 (did not experience the complication in the last 3 months) to 3 (severe or chronic problem over last 3 months). The score for each item was determined by the assessor after asking the participant any question deemed relevant, and after physically examining the participant if necessary. The maximum possible total score of the SCI-SCS is 48, where 0 represents no complications and 48 represents severe complications over the last 3 months.

Pressure ulcers were assessed using the Pressure Ulcer Scale for Healing version 3 (PUSH) [23, 24]. The assessor examined the participant’s skin and rated any pressure ulcers on a scale of 0–17. The rating took into account the area of the pressure ulcer (scored from 0 to 10 using grid paper manufactured for this purpose), amount and type of exudate (scored from 0 [none] to 3 [heavy]), and extent of tissue type (scored from 0 [closed] to 4 [necrotic tissue]). If a participant had more than one pressure ulcer the worst pressure ulcer was assessed.

Depression was assessed using the Bangla version of the Centre for Epidemiologic Studies Depression Scale revised version (CESD-R) [25, 26]. The questionnaire contains 20 items, each scored on a 4-point scale. Each item refers to feelings in the past week. Scores are tallied to a total out of 60. A total CESD-R score of 16 or more is indicative of depression. The questionnaire was administered as a self-reported questionnaire with assistance from the assessor if needed.

Participation was assessed using the Bangla version of the eight participation items of the World Health Organization Disability Assessment Schedule version 2 (WHODAS 2.0) [27].The participant was asked how much of a problem he or she had with each participation domain over the preceding 30 days. Each item is scored on a 5-point scale ranging from none (1 point) to extreme/cannot do (5 points). A total score of 8 represents no problems with community participation and a total score of 40 represents extreme problems with participation. The WHODAS was administered as a self-reported questionnaire with assistance from the assessor if needed.

Health-Related Quality of Life was self-assessed, with assistance from the assessor if needed, using the Bangla version of the Short Form Health Survey-12 (SF12) questionnaire [28, 29]. The SF12 consists of 12 questions each graded on a 2- to 6-point scale designed to measure functional health and well-being from the individual’s perspective. Physical component and mental component summary scores were obtained using a standard algorithm developed from a US general population unadjusted for age and gender. Scores were standardised so that a score of 50 represents average functioning with a SD of 10. Higher scores reflect a better quality of life.

Independence was assessed using the self-report version of the Spinal Cord Independence Measure III (SCIM-SR). This is a 17-item test covering key aspects of independence. It rates self-care (four items), respiration and sphincter management (four items), and mobility (nine items) [30]. The items are scored on scales ranging from 0–1 through to 0–15 points and summed to an overall score out of 100, where a higher score reflects more independence. The assessors determined the score for each item after interviewing participants.

Participants were also asked if they had got out of bed, got out of their homes, and engaged in paid work over the last week. These questions were only asked at the 2-year assessment. The three questions were self-administered with assistance from the assessor if needed. In addition, participants in both groups were asked how often they had been in contact with CRP staff since discharge from the CRP. Detailed cost data were also collected. Participants were asked at the 2-year assessment to estimate spinal cord injury-related out-of-pocket costs they incurred over the preceding 2 years. Cost data will be reported elsewhere [18].

### Trial fidelity

The healthcare professionals providing the intervention were physiotherapists with clinical experience in the management of spinal cord injury. They were provided with a written study manual and trained by the principal investigators and other professionals with extensive experience in the management of spinal cord injury in low- and middle-income countries. Refresher training was provided as needed. Day-to-day support was provided by the trial investigators based in Australia and other countries. Experienced trial monitors from George Clinical, India visited the CRP on eight occasions to audit compliance with the trial protocol and with the International Conference of Harmonisation Harmonised Tripartite Guideline for Good Clinical Practice. There was only one change to the protocol: 13 months after the first participant was randomised, the minimum age for participation in the trial was lowered from 18 years to 15 years to increase the rate of recruitment.

### Statistical analysis

The sample size was informed by our earlier study, which investigated 2-year survival after discharge from CRP in a cohort of 350 people with recent spinal cord injury [4]. A sample size of 410 people (205 in each group) provided 80% power (*α* = 0.05) to detect an increase in survival from 83% to 93% with a two-tailed log rank test allowing for a single interim analysis and a worst-case 15% loss to follow up.

Data were analysed by statisticians from the George Institute for Global Health (including QL) using SAS Enterprise Guide version 7.1 (SAS/Stat version 9.4) and replicated by one of the investigators (RDH) using Stata v16. The analyses were first conducted using dummy-randomised data and then, after discrepancies between the two analyses had been resolved, on the data as randomised. An independent Data Monitoring Committee monitored unblinded outcomes and adverse event data according to a written charter and conducted a formal interim analysis when the first 214 participants had been followed up. The protocol provided an option to terminate the trial early if there were safety concerns but not on the basis of futility.

Data were analysed on an intention to treat basis. All tests were two-sided tests with a critical probability of 5%. The primary analysis compared all-cause mortality in the Intervention and Control groups using the log-rank test. Sensitivity analyses were conducted using a Cox model adjusted for level of lesion, combined tests of restricted mean survival times with and without adjustment for level of lesion (tetraplegia or paraplegia) [31], and tests of the difference in the incidence proportion of deaths at the 2-year assessment with and without adjustment for level of lesion (tetraplegia or paraplegia) using log-binomial regression [17]. The size of the effect of intervention was expressed as hazard ratios, differences and ratios of restricted mean survival times at 2 years, and differences in the incidence proportions of death at the 2-year assessment.

The effects of intervention on secondary outcomes were estimated using linear models adjusted for level of lesion. For continuous outcomes, baseline scores were included in the model to increase precision and provide adjusted estimates. For binary outcomes, log-binomial regression was used to estimate the relative risk.

Cox models with interaction terms were used to examine whether the effect of the intervention on survival was moderated by level of lesion (tetraplegia or paraplegia) or age (<30, 30–50, >50 years).

## Results

**Fig. 1:**
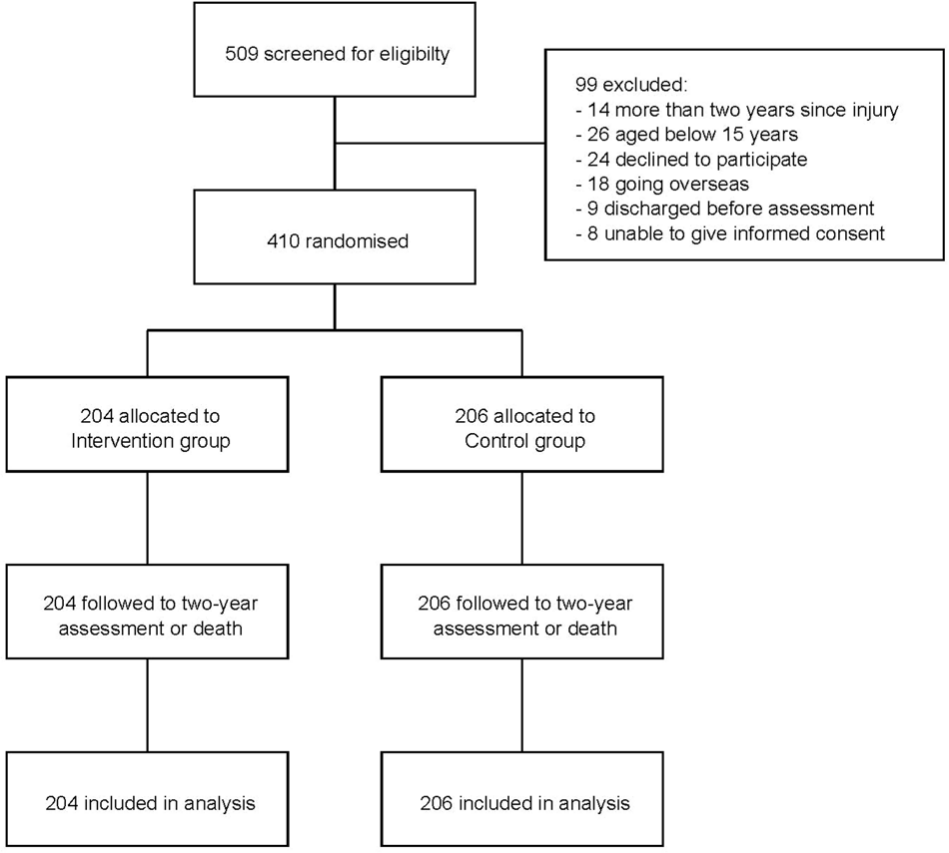
Flow of participants through the trial.

Between 12th July 2015 and 19th March 2018, 509 wheelchair-dependent people with spinal cord injury admitted to CRP were screened for inclusion in the trial. Of these, 75 were ineligible to participate and 24 declined to participate so 410 participants were randomly assigned to the Control (*n* = 206) or Intervention group (*n* = 204; Fig. 1). There were two protocol deviations: two participants were randomised using the wrong stratum. In both cases the error was picked up within a day and the participants were re-randomised using the correct stratum.

The two groups were similar at baseline (Table 1). Two-year outcomes were measured at a median (IQR) of 24.3 months (24.0–24.5) after randomisation. We did not identify any instances of assessor unblinding. All participants were assessed or known to have died at the 2-year assessment, so there was no loss to follow-up. Two participants’ motor scores were not measured at baseline. These are the only missing data.

**Table 1:** Baseline characteristics of participants.

	Control	Intervention
	(*N* = 206)	(*N* = 204)
Age in years		
Median (IQR)	31.4 (24.5 to 41.0)	33.4 (25.7 to 45.0)
Time since injury in months		
Median (IQR)	5.9 (4.6 to 8.2)	5.9 (4.6 to 8.1)
Gender, *n* (%)		
Male	188 (91%)	181 (89%)
Female	18 (9%)	23 (11%)
Cause of injury, *n* (%)		
Traumatic	198 (96%)	192 (94%)
Non-traumatic	8 (4%)	12 6%)
Neurological level of lesion, *n* (%)		
C1 to C4	59 (29%)	61 (30%)
C5 to C8	28 (14%)	23 (11%)
T1 to T7	42 (20%)	34 (17%)
T8 to T12	66 (32%)	80 (39%)
L1 to L5	11 (5%)	6 (3%)
ASIA impairment scale grade, *n* (%)		
A	148 (72%)	144 (71%)
B	34 (17%)	23 (11%)
C	23 (11%)	32 (16%)
D	1 (1%)	5 (3%)
Total motor score/100		
Median (IQR)	50 (27 to 50)^a^	50 (29 to 50)^a^
Marital status, *n* (%)		
Married	132 (64%)	152 (75%)
Never married	62 (30%)	45 (22%)
Separated/divorced	8 (4%)	7 (3%)
Widowed	4 (3%)	0 (0%)
In paid employment prior to injury, *n* (%)		
No	30 (15%)	35 (17%)
Yes	176 (85%)	169 (83%)
Monthly income prior to injury in USD		
Median (IQR)	106.1 (58.9 to 176.8)	94.3 (58.9 to 176.8)
Monthly family income in USD		
Median (IQR)	153.2 (88.4 to 235.7)	153.2 (94.3 to 235.7)
Anticipated primary carer post discharge, *n* (%)		
Spouse	116 (56%)	129 (63%)
Parent	70 (34%)	58 (28%)
Child	4 (2%)	4 (2%)
Other	16 (8%)	13 (6%)

All baseline data were collected prior to randomisation and discharge. 1 US Dollar = 84.86 Bangladeshi Taka, ASIA = American Spinal Injuries Association. ^a^Two motor scores were missing, one from each group.

The intervention was delivered in a way that was generally consistent with the protocol. Participants in the Intervention group received a median (IQR) of 39 (38 to 40) phone calls (the target was 38) and 3.0 (3.0 to 3.0) home visits (the target was 3). The median duration of phone calls was 10 (IQR 9 to 11) min. Participants in the Intervention group reported similar levels of usual care (i.e., care provided after discharge from CRP other than the care provided as part of the trial intervention) to participants in the Control group; the group received, on average, 2 (0 to 5) phone calls, 1 (0 to 1) home visit from CRP staff, and 1 (0 to 5) contact with other healthcare professionals. More details of the intervention and usual care are provided elsewhere [18].

**Fig. 2:**
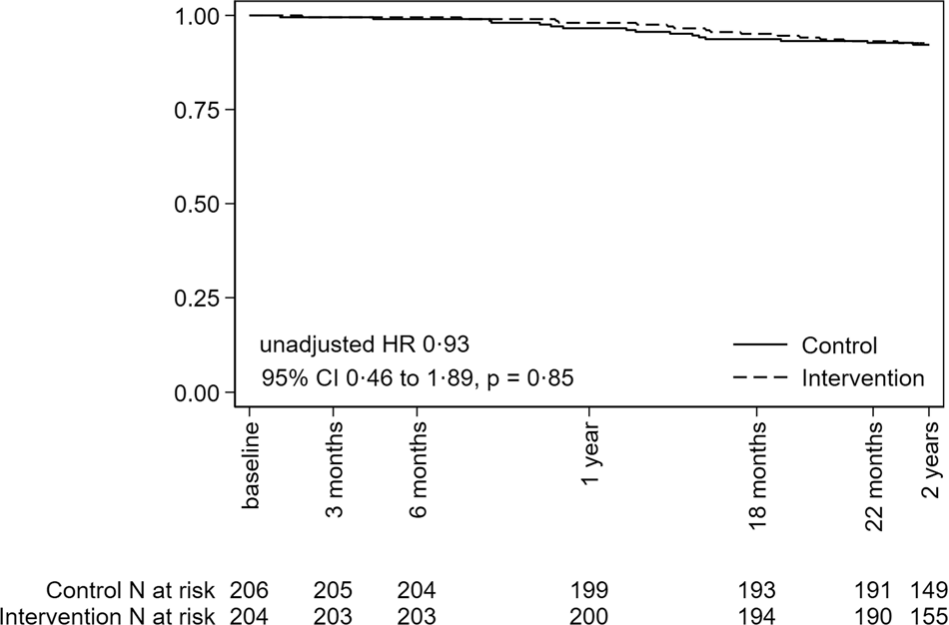
Kaplan–Meier survival curves for the Intervention and Control groups. At 2 years, 15 (7.4%) participants in the Intervention group and 16 (7.8%) participants in the Control group had died. The number at risk is shown below the figure. The numbers at 2 years is less than the number of survivors because some participants were assessed prior to the scheduled 2-year assessment (there was a ±1-month window for assessments to be conducted).

At the 2-year assessment, 15/204 (7.4%) participants from the Intervention group and 16/206 (7.8%) participants from the Control group had died. Figure 2 shows the Kaplan–Meier survival curves. The unadjusted hazard ratio was 0.93 (95% CI 0.46–1.89; *p* value from the log rank test 0.85). None of the sensitivity analyses demonstrated clinically important or statistically significant effects on survival (Table 2). There was no evidence of effect moderation by level of lesion (*p* = 0.51) or age (*p* = 0.44). There were no statistically significant or clinically important differences between groups for any of the continuous secondary outcomes (Table 3) or binary secondary outcomes (Table 4).

**Table 2:** Sensitivity analyses.

Estimand	Method	Estimate (95%CI)	*P* value
Hazard ratio	Cox model, no covariates	0.93 (0.46 to 1.89)	0.85
Hazard ratio	Cox model, adjusted for level of lesion	0.94 (0.47 to 1.91)	0.87
Difference in RMST (months)	Method of Cronin et al. [35]	0.20 (−0.39 to 0.79)	0.51
Difference in RMST (months)	Method of Cronin et al. [35], adjusted for level of lesion	0.21 (−0.38 to 0.80)	0.49
Ratio of RMST	Method of Cronin et al. [35]	1.01 (0.98 to 1.04)	0.51
Ratio of RMST	Method of Cronin et al. [35], adjusted for level of lesion	1.01 (0.98 to 1.04)	0.49
Risk difference	Log binomial regression, no covariates	−0.4% (−5.5% to 4.7%)	0.87
Risk difference	Log binomial regression, adjusted for level of lesion	−0.3% (−5.3% to 4.6%)	0.89

Eight prespecified estimates of the effect of intervention on survival. Hazard ratios were estimated over the 2 years after randomisation. Risk differences were estimated at the 2-year follow-up. RMST = restricted mean survival time at 2 years.

**Table 3:** Continuous secondary outcomes.

	Baseline (SD)		2-year outcome (SD)		Adjusted 2-year outcome (SE)		Adjusted effect (95% CI)	
	Control	Intervention	Control	Intervention	Control	Intervention		*P* value
	(*n* = 206)	(*n* = 204)	(*n* = 189)^a^	(*n* = 189)	(*n* = 189)^a^	(*n* = 189)		
SCI-SCS (/40)	6.0 (2.6)	5.8 (2.8)	7.0 (3.2)	6.7 (2.9)	7.0 (0.2)	6.7 (0.2)	−0.3 (−0.8 to 0.3)	0.39
PUSH (/17)	0.5 (2.0)	0.6 (2.0)	1.4 (3.8)	1.3 (3.5)	1.5 (0.3)	1.3 (0.3)	−0.2 (−0.9 to 0.6)	0.69
CESD-R (/60)	15.9 (9.9)	15.9 (10.1)	17.0 (11.1)	17.0 (10.8)	17.0 (0.8)	17.0 (0.8)	0.0 (−2.1 to 2.1)	1.00
WHODAS (/40)	13.2 (2.8)	13.6 (3.4)	17.9 (5.4)	18.2 (5.3)	18.0 (0.4)	18.2 (0.4)	0.2 (−0.8 to 1.2)	0.69
SF12 PCS	39.9 (4.7)	39.5 (5.4)	36.3 (5.6)	37.0 (6.0)	36.3 (0.4)	37.0 (0.4)	0.7 (−0.3 to 1.8)	0.18
SF12 MCS	48.1 (9.6)	48.4 (9.7)	47.4 (12.9)	47.4 (12.8)	47.5 (0.9)	47.4 (0.9)	−0.1 (−2.6 to 2.4)	0.94
SCIM-SR (/100)	45.0 (19.5)	44.4 (19.0)	50.4 (20.5)	51.4 (19.5)	50.2 (0.8)	51.5 (0.8)	1.3 (−1.0 to 3.6)	0.27

Data are means and SDs, SEs or CIs as indicated. Adjusted outcomes and adjusted effects of intervention (between-group differences) were estimated using linear models that included baseline scores and level of lesion to increase precision. SCI-SCS = Spinal Cord Injury Secondary Conditions Scale (lower scores are better). PUSH = Pressure Ulcer Scale for Healing (lower scores are better). CESD-R = Centre for Epidemiologic Studies Depression Scale revised version (lower scores are better). WHODAS = World Health Organisation Disability Assessment Schedule (lower scores are better). SF12 PCS = Physical component score of the Short Form Health Survey-12 (higher scores are better). SF12 MCS = Mental component score of the Short Form Health Survey-12 (higher scores are better). SCIM-SR = Spinal Cord Independence Measure (higher scores are better). ^a^One participant in the control group was alive 2 years after randomisation but had died by the time his 2-year assessment was conducted.

**Table 4:** Binary secondary outcomes.

	Baseline		2-year outcome		Effect (risk ratio)
	Control	Intervention	Control	Intervention	
	(*n* = 206)	(*n* = 204)	(*n* = 189)^a^	(*n* = 189)	(*n* = 204)
Pressure ulcer	13 (6%)	18 (9%)	27 (14%)	25 (13%)	0.92 (0.56 to 1.53)
Bed-bound	NA	NA	5 (3%)	4 (2%)	0.80 (0.22 to 2.91)
House-bound	NA	NA	54 (29%)	44 (23%)	0.81 (0.58 to 1.14)
Unemployed	NA	NA	139 (74%)	139 (74%)	1.02 (0.92 to 1.13)

Data are number of events (and % of group). Effects of intervention were estimated with log-binomial regression. Risk ratios are adjusted for level of lesion. *NA* not assessed. ^a^One participant in the control group was alive 2 years after randomisation but had died by the time his 2-year assessment was conducted.

To minimise potential for contamination, Control participants were not monitored over the 2-year period. Therefore, there are no data on serious adverse events in the Control group. In contrast, participants in the Intervention group were closely monitored. In this group there were 30 serious adverse events in 25 participants. Six participants developed a serious adverse event deemed life threatening and 19 participants required hospitalisation for 24 serious adverse events. The most common serious adverse events were pressure ulcers (10 serious adverse events in 10 participants) and urinary complications (10 serious adverse events in 8 participants). Causes of death were adjudicated using unblinded data after completion of the trial by two physicians (FB-S and IC) using all available documentation. The most frequent causes of the 31 deaths were pressure ulcers (17 deaths, 55%), suicide or refusal to eat or drink (4 deaths, 13%), and respiratory-related illness (3 deaths, 10%).

## Discussion

These data suggest that a community-based model of care for people with spinal cord injury in Bangladesh did not prevent secondary complications and death in the 2 years after discharge from hospital. We interpret the data in this way because the incidence of deaths was nearly identical in the two groups. The confidence intervals about the primary estimates of effect are quite wide (hazard ratios of 0.46 to 1.89), but sensitivity analyses suggest that if there was any effect of the intervention on survival the effect was small. In particular, the confidence intervals about the ratios of 2-year restricted mean survival (0.98–1.04) and the increase in 2-year restricted mean survival (−0.4 to 0.8 months) suggest clinically important effects are unlikely. Moreover, it would be expected that any effect of intervention would have been mediated by a reduction in the incidence or severity of secondary complications, but there was clearly very little effect of intervention on these outcomes (Table 2). For those reasons we conclude there was not a clinically important effect of the intervention on secondary complications or the risk of death 2 years after discharge.

The 2-year mortality observed in this trial (7.6%) was substantially lower than the 2-year mortality observed in putatively the same population of patients discharged in 2011 (19%) [4]. One explanation could have been that those patients who were eligible to participate in the trial but declined to participate (*n* = 24), or who self-discharged from hospital before trial staff had an opportunity to invite them into the trial (*n* = 9), were more likely to die than trial participants. We followed up these 33 people, after obtaining ethical approval and consent to do so, and found that 12 (36.4%) had died within 2 years of discharge. If these people had been included in the trial the mortality rate across all participants would still have been low (9.7%). In other words, selective recruitment had little effect on the mortality rate. It appears likely, therefore, that mortality rates after discharge from the CRP have decreased since 2011. This could be because better health care is now available to people after hospital discharge, or because there has been a change in the case mix of patients admitted to CRP. A comparison of the baseline characteristics of participants in the earlier cohort study and the current trial suggests that the two cohorts were similar with respect to socioeconomic backgrounds and level of lesion. Regardless of the explanation for the reduction in mortality rates after discharge, the findings of the current trial still hold: the intervention did not prevent secondary complications or death in the first 2 years following discharge.

We had hypothesised that many of the complications people with spinal cord injury commonly develop could be managed at home with appropriate advice and support, and that regular contact with participants, even if only over the phone, would provide an effective way of identifying complications early so that the complications could be managed before becoming insurmountable. The trial findings refute that hypothesis. Similarly, two recent large trials conducted in India and China failed to demonstrate the effectiveness of community-based programs for people with stroke [32, 33]. This highlights the importance of using rigorous research designs to test the effectiveness of community-based interventions that would be widely expected to be effective.

The failure of the intervention to reduce secondary complications and prevent death 2 years after discharge might indicate that the prevention and management strategies recommended by the health professionals were not effective, or that the strategies were not implemented well. Alternatively, it could be that strategies that would otherwise have been effective were ineffective in the current context, even though they were implemented well, because they were administered to people living in poverty with few resources, poor nutrition, and limited access to health care. To the extent that is true, effective long-term intervention for this population may require strengthening of economic and health systems. It is possible that the intervention may have been more effective if delivered by nurses or doctors. We did not employ doctors because of the greater cost. We tried to employ nurses but only one appropriately qualified nurse applied for the position (this person was employed as an assessor). We do not believe that failure to recruit nurses was a major limitation because the physiotherapists were comprehensively trained and became skilled at providing the intervention.

Interestingly, even though healthcare costs for participants in the Intervention group were subsidised (maximum AUD80), this did not improve health outcomes. The small amount of financial assistance may however have gone some way to alleviating the financial strain experienced by participants and their families [34]. There may also be other beneficial effects of the intervention that were not captured with the measured outcomes. As part of a formal process evaluation, 14 participants from the Intervention group were interviewed. All indicated that the regular phone calls alleviated the sense of social isolation and gave them increased confidence to manage their situations [18].

It is possible that the education participants received prior to discharge rendered the post-discharge support unnecessary. During the period of hospitalisation at the CRP, people with spinal cord injury and their carers were educated about prevention and management of secondary complications. Post-discharge phone-based care may be more effective in other contexts where less education is provided while in hospital.

A limitation of this trial was the failure to verify the exact date of death of participants. That occured because Bangladesh does not have a death registry. Participants in the Intervention group were carefully monitored and the dates of deaths were accurately recorded by trial staff. However, there were no equivalent data for the participants in the Control group. To avoid a systematic bias, only dates of death collected by the blinded assessors at 2 years were used for the analyses. The blinded assessors asked families and community members of both groups to report dates of death. These dates may not always have been accurate. However, because the assessors were blinded, it is unlikely that any inaccuracies would have biased the trial’s findings. Another limitation was that cause of death was determined using information reported by families. The data suggest that pressure ulcers were a common cause of death although often it was not certain whether participants died with pressure ulcers or because of pressure ulcers.

The finding that the intervention did not produce clinically important reductions in secondary complications or death 2 years after discharge was disappointing but vindicates the trial. More generally, this finding confirms the importance of assessing effectiveness of health interventions with randomised trials even when there is a strong expectation that the intervention is effective. There remains an urgent need to identify sustainable ways to reduce morbidity and mortality after discharge from hospital with spinal cord injury in low- and middle-income countries.

### Data archiving

Deidentified individual participant data and the accompanying codebook are provided in the Supplementary files.

## Supplementary information

CIVIC data dictionary

CIVIC data file

## References

[1] Zakrasek EC, Creasey G, Crew JD (2015). Pressure ulcers in people with spinal cord injury in developing nations. Spinal Cord.

[2] Bickenbach J, Bodine C, Brown D, Burns A, Campbell R, Cardenas D, et al. International perspectives on spinal cord injury. Geneva: World Health Organization and ISCoS; 2013.

[3] Chamberlain JD, Meier S, Mader L, von Groote PM, Brinkhof MW (2015). Mortality and longevity after a spinal cord injury: systematic review and meta-analysis. Neuroepidemiology..

[4] Hossain MS, Rahman MA, Herbert RD, Quadir MM, Bowden JL, Harvey LA (2016). Two-year survival following discharge from hospital after spinal cord injury in Bangladesh. Spinal Cord.

[5] Hossain MS, Harvey LA, Islam MS, Rahman MA, Glinsky JV, Herbert RD (2018). A prediction model to identify people with spinal cord injury who are at high risk of dying within 5 years of discharge from hospital in Bangladesh. Spinal Cord.

[6] Harnett A, Bateman A, McIntyre A, Parikh R, Middleton J, Arora M, et al. Spinal cord injury rehabilitation practices. In: Eng JJ, Teasell RW, Miller WC, et al., editors. Spinal cord injury rehabilitation evidence. Canada: SCIRE; 2020.

[7] Skelton F, Hoffman JM, Reyes M, Burns SP (2015). Examining health-care utilization in the first year following spinal cord injury. J Spinal Cord Med.

[8] Pagliacci MC, Franceschini M, Di Clemente B, Agosti M, Spizzichino L (2007). and Gisem. A multicentre follow-up of clinical aspects of traumatic spinal cord injury. Spinal Cord.

[9] National Pressure Ulcer Advisory Panel, European Pressure Ulcer Advisory Panel and Pan Pacific Pressure Injury Alliance. Prevention and treatment of pressure ulcers: quick reference guide. Perth, Australia: Emily, H. Cambridge Media; 2014.

[10] Regan MA, Teasell RW, Wolfe DL, Keast D, Mortenson WB, Aubut JA (2009). A systematic review of therapeutic interventions for pressure ulcers after spinal cord injury. Arch Phys Med Rehabil.

[11] Consortium for Spinal Cord Medicine. Bladder management following spinal cord injury: what you should know; a guide for people with spinal cord injury. Paralyzed Veterans of America, Washington (DC). 2011.

[12] Bloemen-Vrencken JH, De Witte LP, Post MW (2005). Follow-up care for persons with spinal cord injury living in the community: a systematic review of interventions and their evaluation. Spinal Cord.

[13] Iemmi V, Gibson L, Blanchet K, Kumar KS, Rath S, Hartley S (2015). Community-based rehabilitation for people with disabilities in low- and middle-income countries: a systematic review. Campbell Syst Rev.

[14] Arora M, Harvey LA, Glinsky JV, Chhabra HS, Hossain S, Arumugam N (2017). Telephone-based management of pressure ulcers in people with spinal cord injury in low- and middle-income countries: a randomised controlled trial. Spinal Cord.

[15] Hossain MS, Harvey LA, Rahman MA, Bowden JL, Islam MS, Taylor V (2016). A pilot randomised trial of community-based care following discharge from hospital with a recent spinal cord injury in Bangladesh. Clin Rehabil.

[16] Hossain MS, Harvey LA, Rahman MA, Muldoon S, Bowden JL, Islam MS (2016). Community-based InterVentions to prevent serIous Complications (CIVIC) following spinal cord injury in Bangladesh: protocol of a randomised controlled trial. BMJ Open.

[17] Herbert RD, Harvey LA, Hossain MS, Islam MS, Li Q, Billot L (2019). Community-based interventions to prevent serious complications following spinal cord injury in Bangladesh: the CIVIC trial statistical analysis plan. Trials..

[18] Liu H, Hossain MS, Islam MS, Rahman MA, Costa PD, Herbert RD, et al. Understanding how a community-based intervention for people with spinal cord injury in Bangladesh was delivered: a process evaluation for the CIVIC trial. Spinal Cord. 2020. 10.1038/s41393-41020-40495-41396. 10.1038/s41393-020-0495-6 PMC7606133 32541882

[19] Consortium for Spinal Cord Medicine. Pressure ulcer prevention and treatment following spinal cord injury; a clinical practice guideline for health-care professionals. 2nd edition. https://www.mascip.co.uk/wp-content/uploads/2015/05/CPG_Pressure-Ulcer.pdf. Accessed 19 Nov 2019. 10.1080/10790268.2001.11753592 11958176

[20] Cameron AP, Wallner LP, Tate DG, Sarma AV, Rodriguez GM, Clemens JQ (2010). Bladder management after spinal cord injury in the United States 1972 to 2005. J Urol..

[21] World Health Organization, UNESCO, International Labour Organization, and International Disability Development Consortium. Community-based rehabilitation: CBR guidelines. 2010; World Health Organization: Geneva.

[22] Kalpakjian CZ, Scelza WM, Forchheimer MB, Toussaint LL (2007). Preliminary reliability and validity of a Spinal Cord Injury Secondary Conditions Scale. J Spinal Cord Med.

[23] Gardner SE, Frantz RA, Bergquist S, Shin CD (2005). A prospective study of the pressure ulcer scale for healing (PUSH). J Gerontol A Biol Sci Med Sci.

[24] Stotts NA, Rodeheaver GT, Thomas DR, Frantz RA, Bartolucci AA, Sussman C (2001). An instrument to measure healing in pressure ulcers: development and validation of the pressure ulcer scale for healing (PUSH). J Gerontol A Biol Sci Med Sci.

[25] Tsutsumi A, Izutsu T, Akramul Islam MD, Amed JU, Nakahara S, Takagi F (2004). Depressive status of leprosy patients in Bangladesh: association with self-perception of stigma. Lepr Rev.

[26] Miller WC, Anton HA, Townson AF (2008). Measurement properties of the CESD scale among individuals with spinal cord injury. Spinal Cord.

[27] Üstün TB, Kostanjsek N, Chatterji S, and Rehm J. Measuring health and disability: manual for WHO Disability Assessment Schedule (WHODAS 2.0). Geneva, Switzerland; 2010.

[28] Islam N, Khan IH, Ferdous N, Rasker JJ (2017). Translation, cultural adaptation and validation of the English “Short form SF 12v2” into Bengali in rheumatoid arthritis patients. Health Qual Life Outcomes.

[29] Feroz AH, Islam MN, ten Klooster PM, Hasan M, Rasker JJ, Haq SA (2012). The Bengali Short Form-36 was acceptable, reliable, and valid in patients with rheumatoid arthritis. J Clin Epidemiol.

[30] Itzkovich M, Shefler H, Front L, Gur-Pollack R, Elkayam K, Bluvshtein V (2018). SCIM III (Spinal Cord Independence Measure version III): reliability of assessment by interview and comparison with assessment by observation. Spinal Cord.

[31] Royston P (2017). A combined test for a generalized treatment effect in clinical trials with a time-to-event outcome. Stata J.

[32] Zhou B, Zhang J, Zhao Y, Li X, Anderson CS, Xie B (2019). Caregiver-delivered stroke rehabilitation in rural China. Stroke..

[33] Lindley RI, Anderson CS, Billot L, Forster A, Hackett ML, Harvey LA (2017). Family-led rehabilitation after stroke in India (ATTEND): a randomised controlled trial. Lancet..

[34] Hossain MS, Harvey LA, Islam MS, Rahman MA, Liu H, Herbert RD (2020). Loss of work-related income impoverishes people with SCI and their families in Bangladesh. Spinal Cord.

[35] Cronin A, Tian L, Uno H (2016). strmst2 and strmst2pw: new commands to compare survival curves using the restricted mean survival time. Stata J.

